# Comparison of three molecular methods for the detection and speciation of *Plasmodium vivax *and *Plasmodium falciparum*

**DOI:** 10.1186/1475-2875-6-124

**Published:** 2007-09-15

**Authors:** Prapaporn Boonma, Peter R Christensen, Rossarin Suwanarusk, Ric N Price, Bruce Russell, Usa Lek-Uthai

**Affiliations:** 1 Department of Parasitology, Faculty of Public Health, Mahidol University, Bangkok, Thailand; 2 International Health Program, Infectious Diseases Division, Menzies School of Health Research and Charles Darwin University, Darwin, Australia; 3 Centre for Vaccinology & Tropical Medicine, Nuffield Department of Clinical Medicine, John Radcliffe Hospital, Oxford, UK

## Abstract

**Background:**

Accurate diagnosis of *Plasmodium *spp. is essential for the rational treatment of malaria. Despite its many disadvantages, microscopic examination of blood smears remains the current "gold standard" for malaria detection and speciation. PCR assays offer an alternative to microscopy which has been shown to have superior sensitivity and specificity. Unfortunately few comparative studies have been done on the various molecular based speciation methods.

**Methods:**

The sensitivity, specificity and cost effectiveness of three molecular techniques were compared for the detection and speciation of *Plasmodium falciparum *and *Plasmodium vivax *from dried blood spots collected from 136 patients in western Thailand. The results from the three molecular speciation techniques (nested PCR, multiplex PCR, and real-time PCR) were used to develop a molecular consensus (two or more identical PCR results) as an alternative gold standard.

**Results:**

According to the molecular consensus, 9.6% (13/136) of microscopic diagnoses yielded false negative results. Multiplex PCR failed to detect *P. vivax *in three mixed isolates, and the nested PCR gave a false positive *P. falciparum *result in one case. Although the real-time PCR melting curve analysis was the most expensive method, it was 100% sensitive and specific and least time consuming of the three molecular techniques investigated.

**Conclusion:**

Although microscopy remains the most appropriate method for clinical diagnosis in a field setting, its use as a gold standard may result in apparent false positive results by superior techniques. Future studies should consider using more than one established molecular methods as a new gold standard to assess novel malaria diagnostic kits and PCR assays.

## Background

In 2005 more than three billion people were at risk of malaria infection [1]. In addition to *Plasmodium falciparum *there are four other malaria species known to infect humans; *Plasmodium vivax*, *Plasmodium ovale*, *Plasmodium malariae *and *Plasmodium knowlesi *[2].

Since *P. falciparum *infections are potentially fatal, quick and accurate diagnosis is vital for the effective treatment of this parasitic disease with artemisinin combination therapy (ACT) [3]. In Thailand, chloroquine remains the first line treatment of *P. vivax *since it is a safe and cost effective anti-malarial and its long half-life protects the patient from the first relapse after treatment. The current "gold standard" for *Plasmodium *spp. speciation is microscopic examination of blood smears [4-8]. Microscopy provides a cost effective, rapid diagnostic tool which can easily be applied in the field. However, even the best malaria microscopists are proned to misdiagnosis, especially in cases of mixed infection and when only tiny rings are present [9]. In 2002, the United Kingdom National External Quality Assessment Scheme for Parasitology cross-checked the results of species identification by microscopists from 262 laboratories and found that the accuracy varied from 64% to 95% [6].

PCR offers an alternative to microscopy which has shown in many cases to have superior sensitivity and specificity [10-12]. There are many techniques which utilise PCR to speciate malaria parasites [9,13,14], though these techniques are rarely evaluated side by side.

The aim of this study was to compare three molecular techniques for the detection and speciation of *P. falciparum *and *P. vivax *from dried blood spots collected from malaria patients in western Thailand. The results from the three molecular speciation techniques; nested PCR [9], multiplex PCR [13] and real-time PCR [14] were compared to microscopy.

## Methods

### Study site and sample collection

The samples for this study were collected between the months of March and October 2006 from volunteers seeking care at malaria clinics in Pong Nam Ron, Chanthaburi Province, near the Thailand-Cambodia border and Suan-Peung, Ratchaburi Province, located on the Thailand-Myanmar border. Blood samples were obtained from all patients presenting with acute malaria symptoms. Approximately 3 × 50 μl of whole blood were collected on 3 mm chromatography paper (Whatman) by finger prick for PCR, and standard Giemsa stained thick and thin blood films prepared in the field. *Plasmodium *infection was determined by a field microscopist and then sent with dry blood samples to Mahidol University, Bangkok. Genomic DNA was extracted from the blood spots using QIAamp^® ^DNA MiniKits, yielding 150 μl of template per spot.

This study was approved by the ethical Committee on Human Rights Related to Human Experimentation, Mahidol University, Bangkok (#15/2004). Samples were only taken after written consent was given and the study was explained in Karen, Myanmese or Thai.

### Multiplex PCR

**Figure 1:**
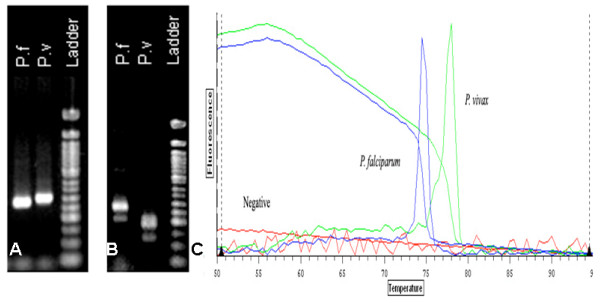
**An example of results from each of the PCR assays examined in this study. The example uses a paired single infection of *Plasmodium falciparum *and *Plasmodium vivax***. A. Image of *P. falciparum *(300-bp) and *P. vivax *(276-bp) positive samples after multiplex PCR, run on 2% agarose at 100 v for 1 hour. B. *P. falciparum *(205-bp) and *P. vivax *(120-bp) positive samples after 2^nd ^round nested PCR, run on 2% agarose at 100 v for 1 hour. C. Real-time PCR melt curves of *P. falciparum *(*T*_*m *_74.5) and *P. vivax *(*T*_*m *_78). Image provided by Optical Monitor Software v.3.1.

Multiplex PCR was carried out using the five primers previously described by Padley *et al *[13] (Table 1). The reaction mix consisted of 12.5 μl of 2× QIAGEN Multiplex PCR Master Mix, 1.5 μl of Reverse, *P. falciparum *and *P. vivax *primers, 2.5 μl of *P. malariae *and *P. ovale *primers, 5 μl of template DNA and 23 μl of RNase free water, making a final reaction volume of 50 μl. Amplification was performed under the following conditions: 95°C for 15 min followed by 43 cycles of 94°C for 45 sec, 60°C for 90 sec and 72°C for 5 min. A multiplex PCR a positive result was recorded when post amplification product run out on agarose via electrophoresis provided a band between 276-bp and 412-bp in length. A 276-bp product indicated infection by *P. vivax*, 300-bp was *P. falciparum *(Figure 1A).

**Table 1:** Primer Sequences

**PCR**	**Primer Name**	**Primer Sequence**
**Nested – 1^st ^round**	rPLU6	TTA AAA TTG TTG CAG TTA AAA CG
	rPLU5	CCT GTT GTT GCC TTA AAC TTC

**Nested – 2^nd ^round**	rFAL1	TTA AAC TGG TTT GGG AAA ACC AAA TAT ATT
	rFAL2	ACA CAA TGA ACT CAA TCA TGA CTA CCC GTC
	rVIV1	CGC TTC TAG CTT AAT CCA CAT AAC TGA TAC
	rVIV2	ACT TCC AAG CCG AAG CAA AGA AAG TCC TTA
	rMAL1	ATA ACA TAG TTG TAC GTT AAG AAT AAC CGC
	rMAL2	AAA ATT CCC ATG CAT AAA AAA TTA TAC AAA
	rOVA1	ATC TCT TTT GCT ATT TTT TAG TAT TGG AGA
	rOVA2	GGA AAA GGA CAC ATT AAT TGT ATC CTA GTG

**Multiplex**	Reverse	GTA TCT GAT CGT CTT CAC TCCC
	PF	AAC AGA CGG GTA GTC ATG ATT GAG
	PV	CGG CTT GGA AGT CCT TGT
	PO	CTG TTC TTT GCA TTC CTT ATG C
	PM	CGT TAA GAA TAA ACG CCA AGC

**Real-time**	Primer 1	TAA CGA ACG AGA TCT TAA
	Primer 2	GTT CCT CTA AGA AGC TTT

Primer sequences for 1^st ^round nested PCR, 2^nd ^round nested PCR, multiplex PCR and real-time PCR.

### Nested PCR

Nested PCR was carried out using the ten primers described by Snounou *et al *[9] (Table 1). The first round of genus specific amplification was carried out in a 20 μl reaction consisting of 2 μl 10× buffer, 1.6 μl of 25 mM MgCl_2_, 0.25 μl of 10 mM dNTPs, 1 μl of 2.5 μM primer rPLU6, 1 μl of 2.5 μM primer rPLU5, 0.08 μl of HotStarTaq, 2 μl of sample DNA and 12.07 μl of double distilled water. Amplification was performed using a under the following conditions: 95°C for 15 min, 25 cycles of 58°C for 2 min, 72°C for 5 min and 94°C for 1 min, followed by 58°C for 2 min and 72°C for 2 min.

The second round of nested PCR was carried out in four separate tubes each containing a single primer pair. The reaction mix contained 2 μl 10× buffer, 1.6 μl of 25 mM MgCl_2_, 0.25 μl of 10 mM dNTPs, 1 μl of each primer in a single pair (2.5 μM), 0.08 μl of HotStarTaq, 1 μl of template DNA and 13.07 μl of double distilled water with a final reaction volume of 20 μl. Amplification was performed under the following conditions: 95°C for 15 min, 30 cycles of 58°C for 2 min, 72°C for 5 min and 94°C for 1 min, followed by 58°C for 2 min and 72°C for 2 min.

Nested PCR amplification product was detected via ethidium bromide staining after 2% agarose gel electrophoresis. A positive reaction is noted when primers for *P. falciparum *and *P. vivax *produce amplification products of 205-bp and 120-bp respectively (Figure 1B).

### Real-Time PCR

Real-time PCR was carried out using the two primers described by Mangold *et al *[14] (Table 1). A 20 μl reaction volume was used consisting of 10 μl of 2× QuantiTect SYBR Green, 4 μl MgCl_2_, 0.1 μl of each primer, 2 μl of DNA template and 3.8 μl of RNase free water. Real-time amplification, speciation was performed using a Chromo4^® ^System (Bio-Rad, U.S.A.) under the following conditions: 95°C for 15 min, 40 cycles of 94°C for 15 sec, 50°C for 30 sec and 72°C for 30 sec. Speciation was determined via a melt programme consisting of stepwise temperature increases of 0.5°C/s starting at 50°C and ending at 95°C with fluorescence acquisition at each temperature transition. The real-time PCR *T*_*m *_values slightly differed to those published by Mangold et al. 2005 [14]. To determine the correct *T*_*m *_values for this study, positive controls were amplified and run through the melt programme.

*T*_*m *_values between 77°C and 78°C indicated infection with *P. vivax *while a *T*_*m *_value between 74°C and 74.5°C indicated infection with *P. falciparum *(Figure 1C). Uninfected RBC negative controls were obtained from donors who have not visited a malaria endemic area.

### Analysis

In order to evaluate molecular speciation against microscopy, a molecular consensus was derived from the collective results of the three PCR assays (Table 2). This molecular consensus was used as an alternative gold standard for sensitivity and specificity analysis. Sensitivity and specificity analyses were recalculated using microscopy as the gold standard.

**Table 2:** Speciation results of all samples

**Percentage of total isolates (n = 136)**	**PCR**			**Molecular Consensus**	**Microscopy**
			
	**Multiplex**	**Nested**	**Real-time**		
23.5% (32)	*Pf*	*Pf*	*Pf*	*Pf*	*Pf*
0.7% (1)	*Pf*	*Pf + Pv*	*Pf + Pv*	*Pf+ Pv*	*Pf*
34.6% (47)	*Pv*	*Pv*	*Pv*	*Pv*	*Pv*
2.2% (3)	Neg	Neg	Neg	Neg	*Pv*
1.5% (2)	*Pf*	*Pf*	*Pf*	*Pf*	*Pv*
1.5% (2)	*Pf*	*Pf*	*Pf*	*Pf*	*Pf + Pv*
1.5% (2)	*Pf*	*Pf + Pv*	*Pf + Pv*	*Pf + Pv*	*Pf + Pv*
30.9% (42)	Neg	Neg	Neg	Neg	Neg
2.2% (3)	*Pv*	*Pv*	*Pv*	*Pv*	Neg
0.7% (1)	*Pf*	*Pf*	*Pf*	*Pf*	Neg
0.7% (1)	*Pv*	*Pf + Pv*	*Pv*	*Pv*	Neg

Results are *P. falciparum *(Pf), *P. vivax *(Pv) or negative (Neg). Samples are grouped by similar results by all techniques, there are 11 result combinations and are represented in each of the tables rows. Molecular consensus in column five is was determined when two or more of the three molecular techniques were alike.

## Results

Of the 136 patients presenting to the clinic with malaria symptoms, only 65.4% (89/136) had a *Plasmodium *spp. infection detected by microscopy. According to microscopic examination 24.3% (33/136) of specimens were positive for *P. falciparum *and 38.2% (52/136) for *P. vivax*. In 2.9% (4/136) of specimens a mixture of both species were detected (Table 2).

In 91 (67%) specimens, all three PCR techniques detected *Plasmodium *spp., two more than by microscopy. In positive specimens the speciation results for the PCR methods concurred in all but 4 (4.4%) cases. All conflicting speciation results were present in samples with a mixed infection result from at least one speciation method. In three of these cases Multiplex PCR did not detect *P. vivax *and in one case the nested PCR sample indicated a mixed infection when the consensus was a *P. vivax *single infection (Table 2).

The sensitivity and specificity results for the four speciation techniques differed dependent upon whether microscopy or molecular consensus was used as the gold standard. Using microscopy as gold standard, the sensitivity of all three molecular techniques for the overall detection of *Plasmodium *spp. was 96.6% (86/86+3) and the specificity was 89.4% (42/42+5) (Table 3B). However, when molecular consensus was used as the gold standard the sensitivity and specificity of microscopy was 94.5% (86/86+5) and 93.3% (42/42+3) respectively (Table 3A).

**Table 3:** A. Sensitivity and specificity of all three molecular techniques and microscopy using molecular consensus as the gold standard; and B. microscopy as the gold standard

A						
**Molecular consensus as gold standard**	***P. falciparum***		***P. vivax***		**Mixed Infection**	
	
	**Sensitivity (%)**	**Specificity (%)**	**Sensitivity (%)**	**Specificity (%)**	**Sensitivity (%)**	**Specificity (%)**

**Multiplex**	100 (40/40+0)	100 (96/96+0)	92.7 (51/51+4)	100 (81/81+0)	0 (0/0+3)	100 (133/133+0)
**Nested**	100 (40/40+0)	99 (95/95+1)	100 (54/54+0)	100 (82/82+0)	100 (3/3+0)	99.2 (132/132+1)
**Real-time**	100 (40/40+0)	100 (96/96+0)	100 (51/51+0)	100 (85/85+0)	100 (3/3+0)	100 (133/133+0)
**Microscopy**	92.5 (37/37+3)	100 (96/96+0)	90.7 (49/49+5)	91.5 (75/75+7)	66.7 (2/2+1)	98.5 (131/131+2)
						
B						
**Microscopy as gold standard**	***P. falciparum***		***P. vivax***		**Mixed Infection**	
	
	**Sensitivity (%)**	**Specificity (%)**	**Sensitivity (%)**	**Specificity (%)**	**Sensitivity (%)**	**Specificity (%)**

**Multiplex**	100 (37/37+0)	97 (96/96+3)	83.9 (47/47+9)	95 (76/76+4)	0 (0/0+4)	100.00 (132/132+0)
**Nested**	100 (37/37+0)	96 (95/95+4)	87.5 (49/49+7)	93.8 (75/75+5)	50 (2/2+2)	98.5 (130/130+2)
**Real-time**	100 (37/37+0)	97 (96/96+3)	87.5 (49/49+7)	93.8 (75/75+5)	50 (2/2+2)	99.2 (131/131+1)

Sensitivity equals the number of true positives divided by the number of true positives and false negatives combined. Specificity equals the number of true negatives divided by the number of true negatives and false positives combined.

Predictably the specificity of all molecular techniques increased when the molecular consensus as gold standard for both *P. falciparum *and *P. vivax*, sensitivity, while sensitivity and specificity of microscopy drops.

The following costs are based on Australian prices and equipment requirements based on the set up at the Menzies School of Health Research (Table 4). Microscopy is the cheapest method in terms of both set up and execution at 0.27 USD per sample with an initial set up cost of approximately 3770 USD for a microscope. The next cheapest speciation technique is multiplex PCR at 6.28 USD per sample followed by nested and real-time PCR at 7.84 and 8.76 USD respectively. The set up costs for any PCR method are high, multiplex and nested require the same equipment which cost 36,028 USD, the major items of value being the gel documentation system and thermal cycler. Real-time PCR is cheaper to set up costing approximately 31,031 USD, the bulk of funds going towards a real-time thermal cycler.

**Table 4:** Comparison of time and cost for conducting each malaria diagnosis assay

**Diagnosis Method**	**Cost Per Sample (USD)**	**Set Up Costs (USD)**	**Preparation & Post amplification (min)**	**Slide examination/or Thermal cycling (min)**	**Total time (min)**
**Microscopy**	0.27	3,770	15	10	25
**Multiplex PCR**	6.28	36,028	240	360	600
**Nested PCR**	7.84	36,028	270	425	695
**Real-time PCR**	8.76	31,031	150	120	270

Prices are for products bought in Australia and Thailand and are converted to US dollars (USD). Prices include all consumables and equipment to carry out the procedures. These are the approximate times required to perform the speciation of a single sample, including DNA extraction for molecular techniques.

Microscopy has the shortest amount of 'hands on' time for a single sample compared to all molecular techniques (Table 4). From DNA extraction to obtaining a result, nested PCR was the longest molecular technique to perform at approximately 11 hours and 30 minutes, real-time PCR was the shortest at four hours and 30 minutes while multiplex PCR took 10 hours.

## Discussion

The debate on the relative merits of microscopy and PCR methods for the detection and speciation of *Plasmodium *spp. infections is not useful since each method has particular advantages which prescribe a specific utility. The latter is dependent upon the rapid cost effective diagnosis in a field setting versus a highly specific and sensitive gold standard for use in malaria research and reference laboratories.

Although this data shows that 9.6% (13/136) of microscopic diagnosis was probably incorrect, microscopy is clearly the only cost effective method for the rapid diagnosis of malaria in a field setting. In contrast, all three PCR methodologies investigated were sensitive, specific and are capable of detecting very low parasitaemia. Although economies of scale can be applied to PCR methods to reduce the time and cost involved in processing each sample, the capital costs and infrastructure needed to run and maintain PCR methods are not practical in most field settings where even intermittent electrical supply is a luxury.

In malaria research and reference laboratories the issue is not whether PCR should be the accepted gold standard, but which method to adopt. This study faced the same problem faced by others when comparing novel malaria detection assays; the traditional gold standard clearly lacks the sensitivity and specificity of the newer assays. The data from this study and data throughout much of the literature, in particular the paper by Coleman *et al *[15] demonstrates that the use of microscopy as gold standard devalues the effectiveness of novel malaria speciation techniques. To counter this a 'molecular consensus gold standard', based on the results of the three PCR assays was developed. Consensus was reached when two or more of the three molecular techniques were alike. Although this is not a perfect solution, it provided an objective way to assess each assay used in this study.

The results for each of the three molecular techniques agreed with the molecular consensus except in four cases. In three of these cases the multiplex failed to detect *P. vivax *in mixed infections and in one case the nested PCR sample gave a mixed infection false positive where the consensus was *P. vivax *single infection. The most likely reason behind the failure of the multiplex to detect *P. vivax *in the mixed infections is that the two PCR products were not visualized as separate bands after electrophoresis since the band sizes for *P. falciparum *and *P. vivax *differ by only 24-bp. These bands could have been separated with increased agarose gel density and/or a longer electrophoresis step. Conducting the multiplex amplifications in separate tubes for each species primer, paired with the reverse genus primer (Table 1) will also improve *P. vivax *detection in mixed species samples, but increase its cost almost fourfold. It should also be noted that the multiplex method uses twice as much template as the nested technique.

The nested PCR false positive for a mixed infection was probably due to contamination in post master mix as there was no amplification in the negative control. Nested PCR is prone to contamination due to the necessity of reagent and product handling before first round PCR, second round PCR and once again before gel electrophoresis. Also, when discussing false positive or negative results from extremely sensitive PCR assays it is important to consider the 'all-or-none' phenomenon. This occurs when attempting to detect *Plasmdodium *spp. in samples with a very low parasitema, where the number of genomes added to the PCR reaction is close to the detection threshold. In these cases it is likely to be sometimes positive and at other times negative when multiple amplifications are run for these samples. Therefore, it is recommended that future studies repeat PCR assays on samples discordant with the molecular consensus.

The 100% sensitivity and specificity of real-time PCR methodology supports its status as the best PCR methodology of the three tested. The rapid melting curve output provides an unambiguous result, without the need for hazardous and time consuming gel electrophoresis. While real-time PCR was the most expensive in regards to consumables, it was the least time consuming of the three PCR assays. Savings in labour costs and an increased sample throughput should offset the increased costs of running the real-time assay.

The scope of this assay is limited by the absence of *P. malariae *and *P. ovale *in the isolates tested. Future comparative studies should incorporate samples from geographical regions endemic for these species, noting that the nested PCR primers used in this study would be inappropriate for the detection of certain *P. ovale *strains in Southeast Asia [16]. As this study only used samples from dried filter paper spots that limit the volume of blood tested, future work should also investigate sample collection methods that allow for a greater potential DNA yield. Such a method would have to be useful in an environment lacking a cold chain. It is hoped that the molecular consensus approach used in this study will provide a more objective method to assess novel malaria diagnostic kits and PCR assays, avoiding the pitfalls of using an anachronistic gold standard.

## Conclusion

Although rapid cost effective microscopy remains the most appropriate method for clinical diagnosis in a field setting, it lacks the sensitivity and specificity to be considered as a gold standard. Using three published molecular methods, a molecular consensus was established, providing an alternative gold standard to assess *Plasmodium *spp. detection and speciation methods. The data from this study suggests that the real-time PCR methodology was the most sensitive and specific method to detect *P. falciparum *and *P. vivax *from clinical blood spots dried on filter paper. The real-time PCR melting curve method is the recommended method for use in malaria reference and research laboratories

## Authors' contributions

PB, PRC and RS processed the isolates, read slides, conducted the PCR methods and helped draft the manuscript. RS also helped troubleshoot and optimise the PCR techniques. RNP participated in conceptualising the method for data analysis and drafting the manuscript. UL and BMR conceived the study, read microscopy slides, participated in its design and coordination and drafting of the manuscript. UL obtained the ethical clearances for this study. All authors read and approved the final manuscript.
